# Metabolomics: Eavesdropping on silent conversations between hosts and their unwelcome guests

**DOI:** 10.1371/journal.ppat.1006926

**Published:** 2018-04-05

**Authors:** Sydney N. Newsom, Laura-Isobel McCall

**Affiliations:** Department of Chemistry and Biochemistry, University of Oklahoma, Norman, Oklahoma, United States of America; University of Wisconsin Medical School, UNITED STATES

## Introduction

Interest in metabolomics has been rising over the past 15 years or more, driven by instrumental and computational advances, complementarity to other “omics” approaches, and usefulness for a variety of applications, including drug development, biomarker discovery, and basic research on pathogen tropism and metabolic potential. This growing interest has been paralleled by increasing applications of metabolomics studies to host–pathogen systems. Signals silently transmitted between host and pathogen via small molecules can be intercepted by researchers using metabolomic techniques for identification and quantification. In this Pearl, we will discuss basic metabolomics principles and examples of their application to the study of microbial pathogenesis.

Metabolomics is the analysis of a complex biological sample to detect and quantify small (approximately 50–1,500 Da), chemically diverse molecular species known as metabolites, including biological molecules (output of core metabolism, secondary metabolites) and externally derived molecules (food additives, drugs, etc.) [1]. They are the outputs and intermediates of enzymatic reactions, as well as their regulators [2]. Metabolites can also regulate gene expression by, for example, direct binding of transcription factors or through upstream signaling pathways [3]. These multifactorial effects are why the metabolome is often considered closest to phenotype [4]. Common metabolomics methods include mass spectrometry (MS)–or nuclear magnetic resonance (NMR) spectroscopy–based approaches. NMR data acquisition is based on the resonance behavior of certain atoms (e.g., ^1^H) in a magnetic field, which is modulated by the surrounding chemical structure [5]. MS separates intact (MS1) or fragmented (MS2, MS/MS, tandem MS) charged particles based on their mass-over-charge ratio (*m/z*). The fragmentation pattern is characteristic of a molecule’s structure [6]. Studies can focus on a list of metabolites (targeted) or on all detectable metabolites under a given analysis setup (untargeted) [5]. Data processing and identification of NMR or MS signals are usually performed using a combination of computational techniques, manual curation, and comparison to authentic standards [1]. However, many of the detected metabolites will have no known matches, making metabolite identification a major challenge in metabolomics [6]. In addition, further comparison with authentic standards is necessary to confirm peak identifications.

Metabolomics in the context of host–pathogen interactions seeks to determine how specific metabolic environments favor pathogen establishment and how metabolite composition varies under infection conditions. For example, metabolomics can be applied to identify biological processes taking place in the host in response to the pathogen or in the pathogen as it adapts and proliferates in host environments. These insights into the conversation between host and pathogen will guide basic research on pathogenesis and provide a foundation for translational studies.

## Investigating host responses using metabolomics

Due to commonalities in core metabolic processes across systems, many core metabolites, such as nucleotides, amino acids or carbohydrates, are structurally identical in host and pathogen and cannot be differentiated using metabolomics techniques [7]. However, because host biomass usually vastly exceeds microbial biomass even under infection conditions, the majority of detected metabolites are expected to be host derived; this assumption has been confirmed by comparing metabolite contents in individual axenic host and pathogen cultures [8] and using spike-in experiments [7].

**Fig 1:**
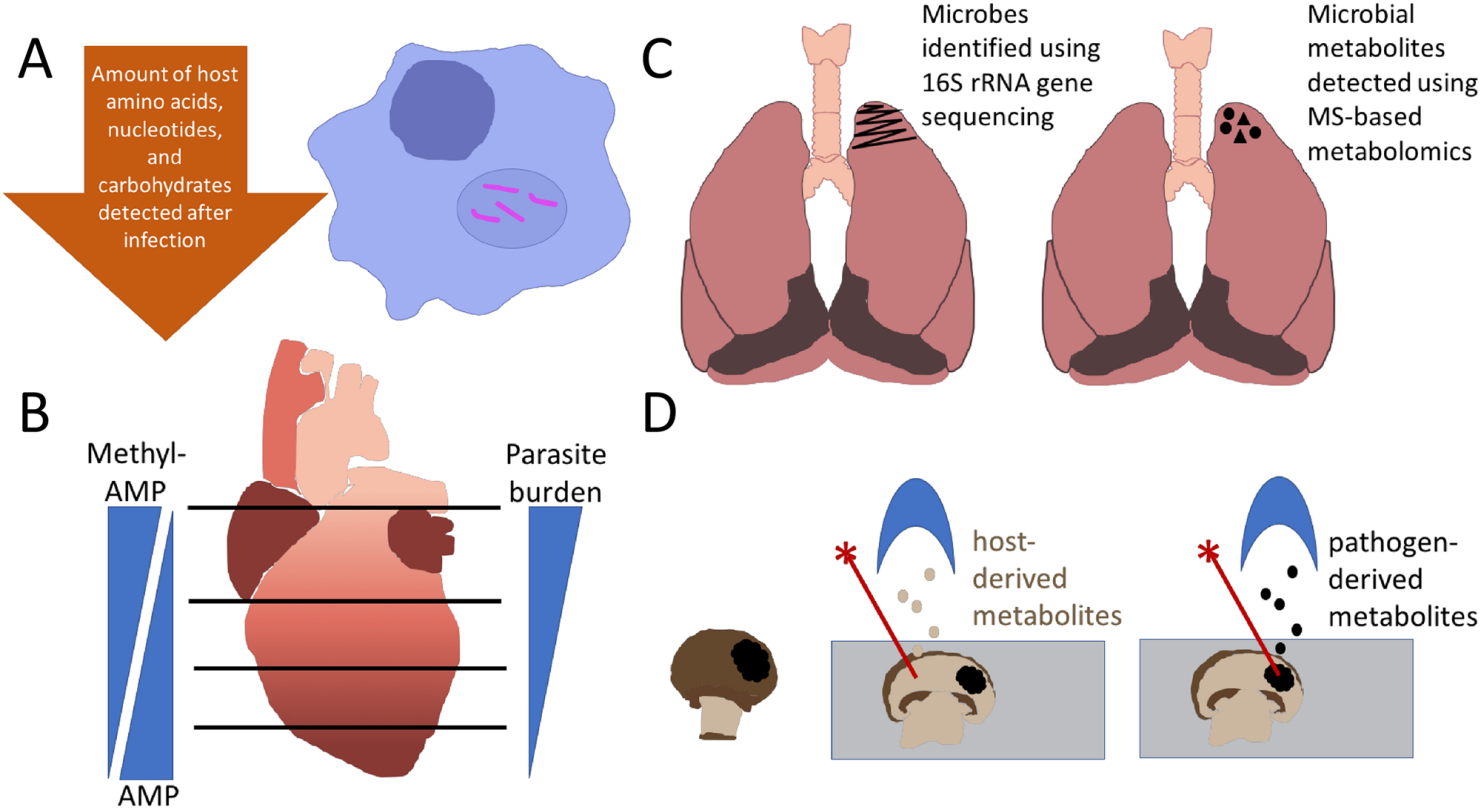
Representative host–microbe metabolomics studies. (A) Rerouting of host metabolism observed in vitro in *M*. *tuberculosis*–infected macrophages [7]. (B) *Trypanosoma cruzi* tropism correlated with host metabolite distribution [9]. (C) Multi-omics approach to study host–pathogen interactions in a diseased human lung [10]. (D) Identification of pathogen-derived molecules using MALDI MS imaging of mushroom tissue within a *Janthinobacterium agaricidamnosum*–infected region [12]. MALDI, Matrix Assisted Laser Desorption Ionization; MS, mass spectrometry.

At the simplest level, metabolomics can be used to study the interaction between pathogen and specific host cell types in an in vitro culture system. For example, MS-based metabolomics of *Mycobacterium tuberculosis*–infected macrophages identified decreases in amino acids, nucleotides, and carbohydrates, reflecting possible consumption by the bacteria (Fig 1A) [7]. Likewise, NMR-based metabolomics of infected cell culture supernatant showed rerouting of host cell metabolism by the intracellular bacterial pathogen *Shigella flexneri* to enable rapid bacterial expansion. These experiments indicated that *Shigella* infection is associated with increased acetate excretion and decreased lactate and pyruvate excretion. Application of these NMR analyses to infection with various *Shigella* metabolic mutants determined that *Shigella* metabolism of host pyruvate is the source of the acetate [8].

Molecular cartography approaches expand these studies to include spatial distribution of metabolites and pathogens. Studies of endogenous metabolite distribution in uninfected samples describe the initial conditions available to the pathogen immediately upon infection in different tissue locations, while investigations of dynamic changes over the course of infection provide information on the changing restrictions on pathogen growth. This approach enabled the identification of preferential parasite distribution to the heart atria and ventricle base in experimental Chagas disease, in association with differential endogenous cardiac metabolite distribution between heart regions (Fig 1B) [9]. Likewise, a comprehensive study of the cystic fibrosis lung showed differential distribution of specific sugars between lung regions (Fig 1C) [10]. These methods provide testable hypotheses to explain preferential pathogen tropism.

## Analyzing microbial signals using metabolomics

Comparison of host–pathogen systems with axenic pathogen cultures or database resources is frequently used to identify pathogen-derived molecules [9–11], with the caveat that many specialized pathogen metabolites are not produced in rich culture conditions [11]. Some metabolites are unique to the pathogen and can be unequivocally assigned a microbial origin. For example, 4,5–9,10-diseco-3-hydroxy-5,9,17-tri-oxoandrosta-1(10),2-diene-4-oic acid (DSHA) is not known to be produced by humans. Its presence in *M*. *tuberculosis*–infected macrophages is due to bacterial degradation of cholesterol [7].

Additional tools include MS imaging, as in a study of the mushroom pathogen *J*. *agaricidamnosum*, which detected the virulence factor jagaricin in infected mushroom tissues [12] (Fig 1D). In contrast, physical separation of host and pathogen prior to metabolomic analysis can enable direct detection of pathogen metabolites. Differential centrifugation followed by MS showed CO_2_ fixation and catabolism of a range of host carbon sources by intracellular *M*. *tuberculosis* [13]. Likewise, in vivo heavy water labeling approaches combined with *Leishmania* isolation from mice showed the relative contribution of salvage and de novo synthesis pathways in pathogen lipid metabolism [14]. The development of new approaches such as coupled Fluorescence-Activated Cell Sorting (FACS)–MS [15] and single-cell metabolomics [16] will advance analysis of trace pathogen metabolites by eliminating host metabolite contamination (Table 1).

**Table 1:** Complementary strengths of discussed metabolomics approaches.

Sample preparation approach	Scale	Strengths for host–pathogen interaction research	Challenges	Examples in host–pathogen research
Profiling of extracts without separation of host and pathogen	Cultured cells, tissue samples	Can be combined with heavy isotope labeling and/or fluxomics for metabolic network and dynamic information Identification of pathogen metabolites if differing from host pathways	No spatial information Limited ability to differentiate between host and pathogen metabolism, especially for common metabolic pathways	[7, 8]
Physical separation of host and pathogen prior to metabolomic analysis (differential centrifugation, FACS, etc.)	Isolated cell populations	Identification and quantification of pathogen-derived metabolites Can be combined with heavy isotope labeling and/or fluxomics for metabolic network and dynamic information	Possibility of artefacts from processing Limit of detection No spatial information	[13, 14]
MS imaging	mm^2^ to cm^2^	Fine-scale spatial information Ability to identify pathogen-derived metabolites by focusing on heavily infected areas	Metabolite identification, unless implemented on instruments with high mass resolution and/or MS/MS capability Usually no dynamic information	[12]
Ex vivo chemical cartography	cm^2^ and above	Large range of surface areas Ability to connect pathogen tissue tropism with metabolite profile	Pathogen is usually not separated from the host tissue prior to analysis, which makes identification of pathogen metabolites more challenging Usually no dynamic information	[9, 10]

Abbreviations: FACS, Fluorescence-Activated Cell Sorting; MS, mass spectrometry.

## Integrative approaches to unravel host and pathogen metabolism

To address the challenge of assigning metabolites to host or pathogen, metabolomics can be combined with other “omics” approaches to differentiate host and pathogen signals and generate comprehensive models of host–pathogen interactions. This is especially important for genome-scale metabolic modeling, as was done in the study of *M*. *tuberculosis*–macrophage interactions [7]. Applying MS tools and temporal sampling to host–pathogen systems fed isotope-labeled nutrients adds dynamic information by enabling differentiation between increased production and decreased consumption of a given metabolite. Such a “fluxomic” approach showed, for example, increased central carbon metabolic flux and increased efflux from the tricarboxylic acid (TCA) cycle to fatty acid biosynthesis during human cytomegalovirus (HCMV) infection [17]. Analysis of labeling patterns also provides information on metabolic network structure, rerouting of pathways (as shown in *Shigella* infection [8]), and on the relative contribution of pathogen de novo versus salvage pathways, as was performed for fatty acid metabolism in *L*. *mexicana* infection [14].

Different “omics” approaches also provide complementary information. For example, fractionation of serum followed by MS analysis identified host serum lysophosphatidylcholine 16:0 (lysoPC [16:0]) as a repressor of *Plasmodium falciparum* asexual to sexual stage differentiation. In vitro transcriptomic analyses enabled identification of downstream responses to lysoPC depletion, including induction of compensatory metabolic pathways and of regulators of parasite differentiation, and metabolomic analysis confirmed lysoPC depletion in vivo during infection [18]. Metabolomic–transcriptomic analyses also helped clarify the pathogenic role of *Saccharomyces cerevisiae* in colitis by revealing lower expression of tight junction–associated genes and increased host purine degradation associated with elevated colon damage in *S*. *cerevisiae*–monocolonized mice [19]. Combining metabolomic and microbiome studies provides further insight into microbiome dynamics and their role in infectious disease pathogenesis. Microbiome, proteomic, and metabolomic studies of fecal samples from *Salmonella enterica* serovar Typhimurium–infected mice showed concurrent proliferation of *S*. *enterica*, mouse immune response induction, depletion of gut commensals, and increase in the sugars they normally metabolize [20]. Garg et al. layered a 16S amplicon sequencing component into their metabolomic model of a diseased lung. Overlaying the metabolite concentrations and microbial species information placed the regional metabolic signaling responses in the context of the varying microbial populations [10].

## Outlook

Metabolomic analyses enable researchers to detect the molecular signals exchanged between hosts and microbes. The interpretation of these silent conversations provides fundamental insight into host–pathogen interactions, which can lead to translational applications. For example, identification of divergent essential pathogen metabolic pathways yields new targets for antimicrobial drug development [21]. New therapies can also be developed to target host pathways critical for pathogen establishment [17]. Likewise, metabolomics is now a key player in biomarker discovery. These tools can be used to facilitate diagnosis of diseases that only present nonspecific clinical symptoms by, for example, assessing changes in bile acids and steroids in febrile illness [22]. In cases in which only a subpopulation of infected individuals progress to severe disease, metabolite signals can be used for patient prognosis [9, 23]. Metabolites are also increasingly being investigated as predictors of treatment success [24] or vaccine efficacy [25]. As metabolomic techniques become more accessible, we expect that they will be used to study a broader range of pathogenic systems as well as polymicrobial infections. New methods to separate host and pathogen metabolites, increased focus on in vivo systems, and collection of dynamic metabolomic information will lead to improved understanding of pathogenesis, with metabolomics bridging the divide between genotype and phenotype.
